# Differential Expression of microRNAs in *Francisella tularensis*-Infected Human Macrophages: miR-155-Dependent Downregulation of MyD88 Inhibits the Inflammatory Response

**DOI:** 10.1371/journal.pone.0109525

**Published:** 2014-10-08

**Authors:** Sarmistha Bandyopadhyay, Matthew E. Long, Lee-Ann H. Allen

**Affiliations:** 1 Inflammation Program, University of Iowa, Coralville, Iowa, United States of America; 2 Department of Internal Medicine, University of Iowa, Iowa City, Iowa, United States of America; 3 Graduate Training Program in Molecular and Cellular Biology, University of Iowa, Iowa City, Iowa, United States of America; 4 Veteran's Administration Medical Center, Iowa City, Iowa, United States of America; Albany Medical College, United States of America

## Abstract

*Francisella tularensis* is a Gram-negative, facultative intracellular pathogen that replicates in the cytosol of macrophages and is the causative agent of the potentially fatal disease tularemia. A characteristic feature of *F. tularensis* is its limited proinflammatory capacity, but the mechanisms that underlie the diminished host response to this organism are only partially defined. Recently, microRNAs have emerged as important regulators of immunity and inflammation. In the present study we investigated the microRNA response of primary human monocyte-derived macrophages (MDMs) to *F. tularensis* and identified 10 microRNAs that were significantly differentially expressed after infection with the live vaccine strain (LVS), as judged by Taqman Low Density Array profiling. Among the microRNAs identified, miR-155 is of particular interest as its established direct targets include components of the Toll-like receptor (TLR) pathway, which is essential for innate defense and proinflammatory cytokine production. Additional studies demonstrated that miR-155 acted by translational repression to downregulate the TLR adapter protein MyD88 and the inositol 5′-phosphatase SHIP-1 in MDMs infected with *F. tularensis* LVS or the fully virulent strain Schu S4. Kinetic analyses indicated that miR-155 increased progressively 3-18 hours after infection with LVS or Schu S4, and target proteins disappeared after 12–18 hours. Dynamic modulation of MyD88 and SHIP-1 was confirmed using specific pre-miRs and anti-miRs to increase and decrease miR-155 levels, respectively. Of note, miR-155 did not contribute to the attenuated cytokine response triggered by *F. tularensis* phagocytosis. Instead, this microRNA was required for the ability of LVS-infected cells to inhibit endotoxin-stimulated TNFα secretion 18–24 hours after infection. Thus, our data are consistent with the ability of miR-155 to act as a global negative regulator of the inflammatory response in *F. tularensis*-infected human macrophages.

## Introduction

*Francisella tularensis* is a facultative intracellular Gram-negative bacterium that causes the zoonotic disease tularemia [1]. This organism infects over 200 types of animals in nature and can be transmitted to humans through insect bites, ingestion of contaminated food and water, or direct contact with infected animals or aerosolized bacteria. Indeed, inhalation of as few as 10 organisms of the highly virulent *F. tularensis* subspecies *tularensis* (type A) strains can be lethal to otherwise healthy individuals, whereas infection with *F. tularensis* subspecies *holarctica* (type B) is typically less severe [1], [2]. Due to its high infectivity, ease of dissemination, and potential lethality, *F. tularensis* is considered a candidate bioweapon. Both type A and type B strains of this pathogen have been classified as Tier 1 select agents, and their possession and study are tightly regulated [1], [3]. An attenuated type B strain was derived several decades ago, but this live vaccine strain (LVS) is not currently licensed for use in the United States [4]. LVS retains many key features of virulent type A and type B *F. tularensis* during *in vitro* infection of eukaryotic cells but does not require biosafety level-3 (BSL-3) containment, and for this reason is an attractive model for studies of tularemia pathogenesis [1], [4], [5].

*F. tularensis* infects several cell types, but macrophages are the major site of bacterial replication *in vivo* and also act as vehicles for bacterial dissemination from the site of infection to the liver and spleen [6]–[8]. Several receptors can mediate *F. tularensis* phagocytosis by macrophages, including the mannose receptor, scavenger receptor A, and complement receptors 3 and 4 (CR3, CR4) [4], [9]. Immediately after uptake *F. tularensis* resides in a phagosome, but within a few hours the phagosome membrane is disrupted by an unknown mechanism, and bacteria escape into the host cell cytosol where they replicate to high density [4], [5], [10].

A distinguishing feature of tularemia is a profound suppression of the host inflammatory response during the first few days of infection, which favors bacterial dissemination and growth and is characterized by diminished proinflammatory cytokine production [4], [11]–[13]. The molecular mechanisms that account for this host defense defect are incompletely defined and are an area of active investigation. Toll-like receptors (TLRs) are critical components of innate defense that detect conserved microbial molecules and initiate downstream signaling which culminates in NF-κB-dependent production of proinflammatory cytokines, including TNFα [14]. Typically, binding of LPS to MD-2/TLR4 complexes allows rapid and sensitive detection of Gram-negative bacteria. However, *F. tularensis* LPS has an unusual structure that impairs its interactions with LPS binding proteins, including MD-2/TLR4 [4], [15]. In addition, LPS O-antigen and capsular polysaccharides protect *F. tularensis* from complement-mediated lysis [1], [16]. Detection of *F. tularensis* is mediated instead by interactions of TLR2 complexes with bacterial lipoproteins [4]. Nonetheless, this reliance on TLR2-dependent signaling does not, in and of itself, account for the impaired host response to this pathogen.

MicroRNAs are small noncoding RNAs that regulate eukaryotic gene expression by base pairing with the 3′untranslated regions (UTR) of their target mRNAs resulting in mRNA degradation and/or translational repression [17]. According to current estimates, the human genome encodes thousands of microRNAs (miRBase: http://www.mirbase.org) targeting ∼60% of all protein-coding genes [18]. A single microRNA can target multiple mRNA species, and a given mRNA is often targeted by multiple different microRNAs, thus leading to a cell- and context-specific network of microRNA-mRNA interactions and outcomes [17]–[19]. MicroRNAs contribute to regulation of most biological processes and also influence numerous pathological states, including cancer and infectious disease [20], [21]. For example, it is now well documented that changes in host microRNA expression regulate the immune response following infection with both Gram-negative and Gram-positive bacteria and act, at least in part, to protect against overwhelming inflammation and sepsis [22]–[26].

*F. novicida* is an environmental organism that does not cause disease in healthy humans or other animals in nature [27] and is more proinflammatory than *F. tularensis* [4], [28], but shares with this pathogen an ability to replicate in mononuclear phagocytes *in vitro* [4]. Published data suggest that miR-155-dependent downregulation of SH2-domain containing inositol phosphatase (SHIP-1), an enzyme that inhibits phosphatidylinositol 3-kinase (PI3K)/Akt signaling by catalyzing the conversion of PI(3,4,5)P_3_ to PI(3,4)P_2_, enhances cytokine production during *F. novicida* infection [29]. In contrast, miR-155 induction is less robust in monocytes infected with the type A *F. tularensis* strain Schu S4, and in this study did not lead to SHIP-1 depletion [29].

Besides the low bioactivity of its LPS, how *F. tularensis* inhibits proinflammatory cytokine production is uncertain, and the full extent to which other miR-155 targets or other microRNAs contribute to this process is unknown. We undertook the present study to address this knowledge gap, and used an unbiased screening strategy to identify microRNAs that were differentially expressed in primary human macrophages infected with *F. tularensis* LVS. Here we report significant differential expression of several microRNAs, including miR-155 and miR-146, and demonstrate that both LVS and Schu S4 ablate expression of MyD88 and SHIP-1 via miR-155-dependent translational repression. At the same time our data indicate that miR-155 induction does not alter the weak inflammatory response that ensues immediately after *F. tularensis* uptake, but contributes instead to the inability of infected cells to be activated upon subsequent stimulation with TLR ligands such as *E. coli* LPS. Therefore, our findings demonstrate that miR-155 plays an anti-inflammatory role in this system and contributes to active inhibition of host defense.

## Materials and Methods

### Bacterial strains and culture conditions

Bacterial strains used in this study include *F. tularensis* subspecies *tularensis* (type A) strain Schu S4, *F. tularensis* subspecies *holarctica* (type B) strain LVS, and *F. novicida* strain U112. Schu S4 and LVS were obtained from Dr. Michael Apicella (University of Iowa, Iowa City, IA), and U112 was obtained from Dr. Colin Manoil (University of Washington, Seattle, WA). All manipulations of virulent *F. tularensis* strain Schu S4 were performed in a licensed BSL-3 facility in the University of Iowa Carver College of Medicine with Select Agent approval and in accordance with all Centers for Disease Control and Prevention and National Institutes of Health regulatory and safety guidelines. Bacteria were grown routinely on cysteine heart agar (Difco, Sparks, MD) plates supplemented with 9% defibrinated sheep blood (Remel, Lenexa, KS) (hereafter called CHAB) for 24–48 h at 37°C in 5% CO_2_. Organisms collected from CHAB plates were resuspended in Hank's Balanced Salt Solution (HBSS) (Cellgro Mediatech, Inc. Manassas, VA) and used to inoculate pH 6.8 brain heart infusion broth (Difco). Broth cultures were grown overnight at 37°C with shaking, and bacteria were harvested during mid-logarithmic phase growth. *Francisella* were collected by centrifugation, washed twice with HBSS, and quantified by measurement of the optical density at 600 nm. For certain experiments, formalin- killed (FK) bacteria were prepared by incubating washed LVS in 10% neutral buffered formalin (Sigma Aldrich, St. Louis, MO) for 45 min at 37°C. FK bacteria were washed three times with HBSS containing divalent cations, and aliquots were plated on CHAB to confirm loss of viability.

### Preparation and infection of human macrophages

Heparinized venous blood was obtained from healthy adult volunteers using protocols approved by the University of Iowa Institutional Review Board for Human Subjects, and all donors provided informed consent. Peripheral blood mononuclear cells (PBMCs) were isolated on Ficoll-Hypaque density gradients and then cultured in sterile screw-cap Teflon jars in HEPES-buffered RPMI-1640 medium supplemented with L-glutamine (Lonza, Allendale, NJ) and 20% autologous human serum at 37°C, 5% CO_2_ for 5 days to allow differentiation of monocytes into monocyte-derived macrophages (MDMs) [10]. The day before infection, cells were recovered from the Teflon jars, washed, resuspended in HEPES-buffered RPMI-1640 medium containing L-glutamine and 10% pooled human serum (PHS), plated in tissue culture dishes, and incubated overnight to allow cell attachment and spreading. The next day, MDM monolayers were washed to remove any nonadherent cells and then infected with bacteria at a multiplicity of infection (MOI) of 100∶1 in fresh medium containing 10% PHS. After 1 h at 37°C, MDMs were washed twice to remove uningested bacteria, and then maintained at 37°C for the indicated amounts of time in medium supplemented with 2.5% PHS. Thereafter, cells were processed for microscopy, RNA isolation, or other endpoints as described below. To quantify bacterial uptake and growth, MDM monolayers were lysed with 0.5% saponin at the indicated time points, and samples were plated on CHAB for enumeration of colony forming units (CFU) after serial dilution. Replicate experiments were performed using MDMs obtained from different donors.

Some experiments utilized THP-1-xBlue-def-MyD88 cells and the corresponding control THP-1-xBlue cells (InvivoGen, San Diego, CA). These human mononuclear cells were maintained in HEPES-buffered RPMI-1640 supplemented with 10% heat-inactivated FBS (HyClone Thermo Scientific, Logan, UT) according to manufacturer's recommendations, differentiated into macrophages by treatment with phorbol myristate acetate (100 nM, 48 h), and then infected with LVS as described above.

### RNA isolation

MDMs were plated in 6-well dishes at 1×10^6^ cells/well and infected as described above. Total macrophage RNA was isolated using Trizol (Invitrogen/Life Technologies, Grand Island, NY) or mirVana RNA isolation kits (Ambion/life technologies, Grand Island, NY) according to the manufacturer's protocols. The quantity of total RNA was measured using a Nanodrop spectrophotometer (Thermo Scientific, Pittsburg, PA), and RNA quality was determined by gel electrophoresis or using a bioanalyzer (Agilent Technologies, Santa Clara CA). All RNAs were stored at −80°C.

### microRNA expression profiling

The expression of 384 human microRNAs was analyzed by real-time PCR using TaqMan human microRNA Arrays A v2.0 microfluidic cards (Applied Biosystems, Life Technologies, Grand Island, NY) as directed by the manufacturer. In brief, total RNA (600 ng) obtained from control MDMs and cells infected with *F. tularensis* LVS for 18 h at an MOI of 100∶1 in three independent experiments were first reverse-transcribed with the Multiplex RT pool set (Applied Biosystems) through a reverse transcription (RT) step using the High-Capacity cDNA Archive Kit (Applied Biosystems), wherein a stem-loop RT primer specifically binds to its corresponding microRNA and initiates its RT. The RT products were subsequently amplified with sequence-specific primers using a 7900 HT Real-Time PCR system (Applied Biosystems). The data were collected and processed using RQ Manager 1.2 and Data Assist v3.0 software (Applied Biosystems). microRNAs with a Ct value ≤35 were included in the analysis and data were normalized to the endogenous control RNU48. microRNA expression fold changes were calculated by the 2^−ΔΔCT^ method [30], and microRNAs with a fold change ≥1.5 and with a *P* value ≤0.05 were classified as significantly differentially regulated.

### Real-time PCR

Mature microRNA expression was quantified using TaqMan microRNA assays (ABI, Life Technologies, Grand Island, NY). Total RNA (10 ng) was reversed transcribed using microRNA specific primers and the TaqMan Reverse Transcription Kit (ABI, Life Technologies). TaqMan microRNA assays were performed on the ABI 7000 Realtime PCR system, using the TaqMan Universal PCR Master Mix (ABI, Life Technologies). RNU48 was used as the internal control, and microRNA expression levels were quantified using the 2^−ΔΔCT^ method.

To analyze mRNA abundace, 150–200 ng of total RNA was reverse transcribed using the High Capacity RT kit (ABI, Life Technologies) and quantitative real-time PCR was performed using gene specific primers and 2× SYBR Green PCR master mix (ABI, Life Technologies). Melt curve analysis was performed at the end of every qRT-PCR run. Relative expression values were quantified using the 2^−ΔΔCT^ method and were normalized to the housekeeping gene glyceraldehyde 3-phosphate dehydrogenase (GAPDH). Fold changes were calculated relative to the untreated control.

### miR-155 overexpression and inhibition

MDMs were plated in 12-well dishes at 1.5×10^5^ cells/well and were transfected the next day with 50 nM miR-155 mimic (Ambion, Austin, TX) or 100 nM miR-155 inhibitor (Dharmacon, Thermo Scientific) along with the negative control pre-miR or anti-miR using SiPORT-NeoFx transfection reagent (Ambion, Austin, TX) in complete medium. Macrophages were infected with bacteria, stimulated with 100 ng/ml *E. coli* O111:B4 LPS (Sigma-Aldrich), or subjected to other treatments 48 h after transfection.

### Immunoblotting and ELISAs

For immunoblotting, cells were resuspended in ice-cold lysis buffer containing phosphatase and protease inhibitors (sodium fluoride, sodium orthovanadate, leupeptin, aprotinin and phenylmethylsulfonyl fluoride) [31], incubated for 5 min at 4°C, and then centrifuged for 10 min to pellet any insoluble cell debris. For some experiments, proteins were isolated from Trizol after RNA extraction as described previously [32]. Protein concentrations of the cleared lysates were measured using the BCA assay (Pierce, Thermo Scientific, Rockford, IL). Equal amounts of total protein (10-15 µg) were separated by SDS-PAGE on 4–12% gradient gels (NuPAGE Bis-Tris pre-cast gels, Life Technologies) and then transferred to polyvinylidene fluoride membranes. After blocking with 5% nonfat dry milk in pH 7.4 TBS with 0.05% Tween 20, membranes were probed with anti-MyD88 or anti-SHIP-1 antibodies (Cat# sc-11356, Cat# sc-6244, Santa Cruz Biotechnology, Dallas, TX), and β-actin (antibody Cat # CP01, Calbiochem, Darmstadt, Germany) was used as the loading control. Bands were detected using horseradish peroxidase-conjugated secondary antibodies (from Bio-Rad, Hercules, CA or Amersham GE HealthCare, Pittsburg) and Super Signal West Pico chemiluminescence reagents (Pierce, Thermo Scientific, Rockford, IL). Blots were exposed to X-ray film or analyzed using a LI-COR Odyssey imaging system (LI-COR Biosystems, Lincoln, NE). For analysis of secreted cytokines, cell-free culture supernatants were collected at the indicated time points after infection or transfection and stored at −80°C. TNFα and IL-6 were quantified by ELISA (eBiosciences, San Diego, CA) with appropriate standard curves run in parallel as previously described [31], [33].

### Flow Cytometry

Viability of MDMs that were transfected with miR-155-specific pre-miRs or negative control pre-miRs for 48 h and then treated with 40 µM LY294002 (Sigma-Aldrich) was assessed using propidium iodide staining and quantiifed by flow cytometry using an Accuri C6 flow cytometer (BD Accuri Cytometers, Inc., Ann Arbor, MI) as we described [9], [31].

### Statistical Analysis

Statistical analysis was performed using GraphPad Prism v.4 or v.6 software, and *P* <0.05 was considered significant by Student's *t* test or analysis of variance with Tukey's post-test for multiple comparisons.

## Results

### Differential expression of microRNAs in LVS-infected MDMs

To determine the extent to which microRNAs were differentially expressed in human macrophages during *F. tularensis* infection, we isolated RNA from control cells and MDMs that were infected with LVS at an MOI of 100∶1 for 18 h, with parallel samples analyzed by microscopy to assess infection efficiency and bacterial burden. We chose the 18 h time point as at this stage of infection bacterial replication is robust, but has not yet affected MDM viability or morphology as evident by microscopy (Fig. S1A). RNA expression profiling was performed in triplicate using TaqMan Low Density Array (TLDA) cards and macrophages from three different donors. Among the 384 human microRNAs analyzed, 269 were either undetectable or below the background, and expression of the remaining 115 microRNAs was further analyzed. Of these, most were unaffected by LVS, and only 10 microRNAs exhibited significant differential regulation defined as >1.5 fold increase or decrease in abundance and *P*<0.05 (Table 1). These data indicate that microRNA expression is significantly altered by LVS infection of human macrophages.

**Table 1:** Differential expression of microRNAs in MDMs infected with *F. tularensis* LVS.

microRNAs	Fold changes	*P* values
**Down Regulated**		
hsa-miR-197-4373102	0.3500	0.0134
**Up Regulated**		
hsa-miR-133a-4395357	1.5348	0.0017
hsa-miR-29c-4395171	1.6746	0.0226
hsa-miR-886-5p-4395304	1.6768	0.0417
hsa-miR-324-3p-4395272	1.6897	0.0349
hsa-miR-146a-4373132	1.7693	0.0181
has-miR-155-4395459	1.9780	0.0332
hsa-miR-126-4395339	2.0521	0.0186
hsa-miR-150-4373127	2.1002	0.0284
hsa-miR-361-5p-4373035	2.4114	0.0268

Differential expression analysis of microRNAs was carried out by TLDA (Applied Biosystems) using control MDMs and cells that were infected with LVS for 18 h. Average fold induction and *P* values were obtained from analysis of triplicate experiments. microRNAs that showed a fold change greater than 1.5 and *p*≤0.05 are shown.

### miR-155 is upregulated by *Francisella* infection

**Figure 1:**
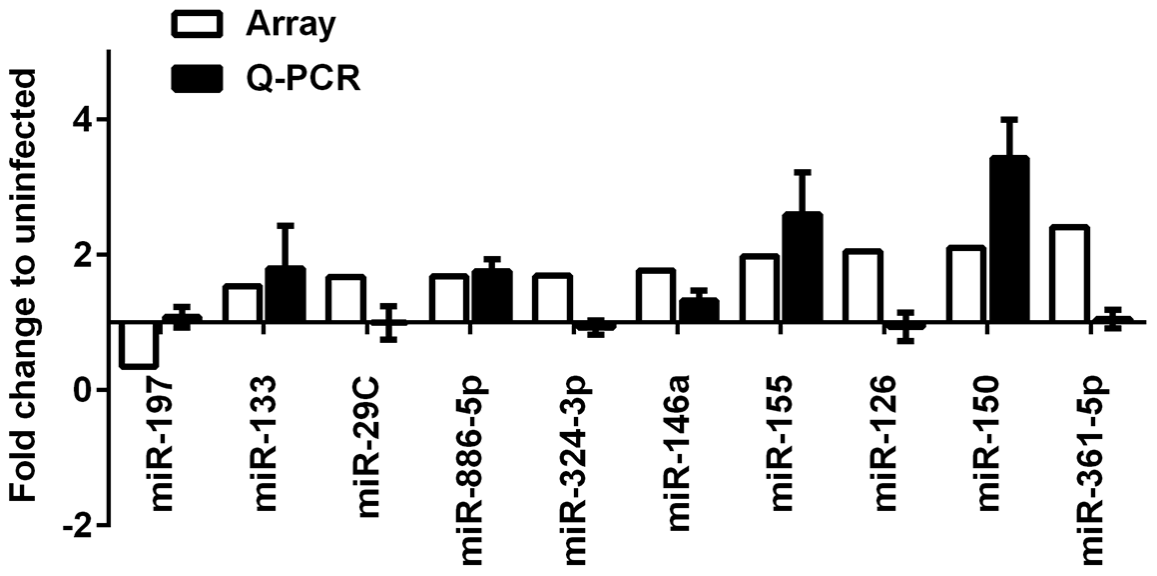
Validation of the TLDA results by qRT-PCR analysis. The relative abundance of differentially expressed microRNAs was determined using individual Taqman microRNA assays, and results were normalized to the internal control RNU48. Data shown are the mean ±SEM from four independent experiments.

Individual Taqman microRNA assays (qRT-PCR based) were performed on the 10 significantly altered microRNAs to validate the results from the screen (Fig. 1). In general, the expression values generated by PCR-array and individual qRT-PCR assays showed consistent results for most of the tested microRNAs from all three sample sets. The correlation coefficient for each validated microRNAs measured by these two approaches was statistically significant (Pearson r = 0.79, *P* = 0.03). Among the altered microRNAs by TLDA analysis, only miR-197 was significantly downregulated, and miR-361-5p was the most upregulated microRNA as judged by differential expression, but this marked change was not validated by individual qRT-PCR assays. In contrast, miR-155, miR-146a and miR-150 were significantly upregulated in infected MDMs as judged by both assays (Fig. 1). These microRNAs are of interest due to their established role in immune regulation, including myeloid cell function and activation state [34]. We focused our studies on miR-155 to define further its role in regulating the macrophage inflammatory response as it has been extensively studied [35], is known to have both pro- and anti-inflammatory effects [36], and because the results of previous studies suggested that this microRNA is markedly induced in mononuclear phagocytes by *F. novicida* but not by *F. tularensis* strain Schu S4 [29].

**Figure 2:**
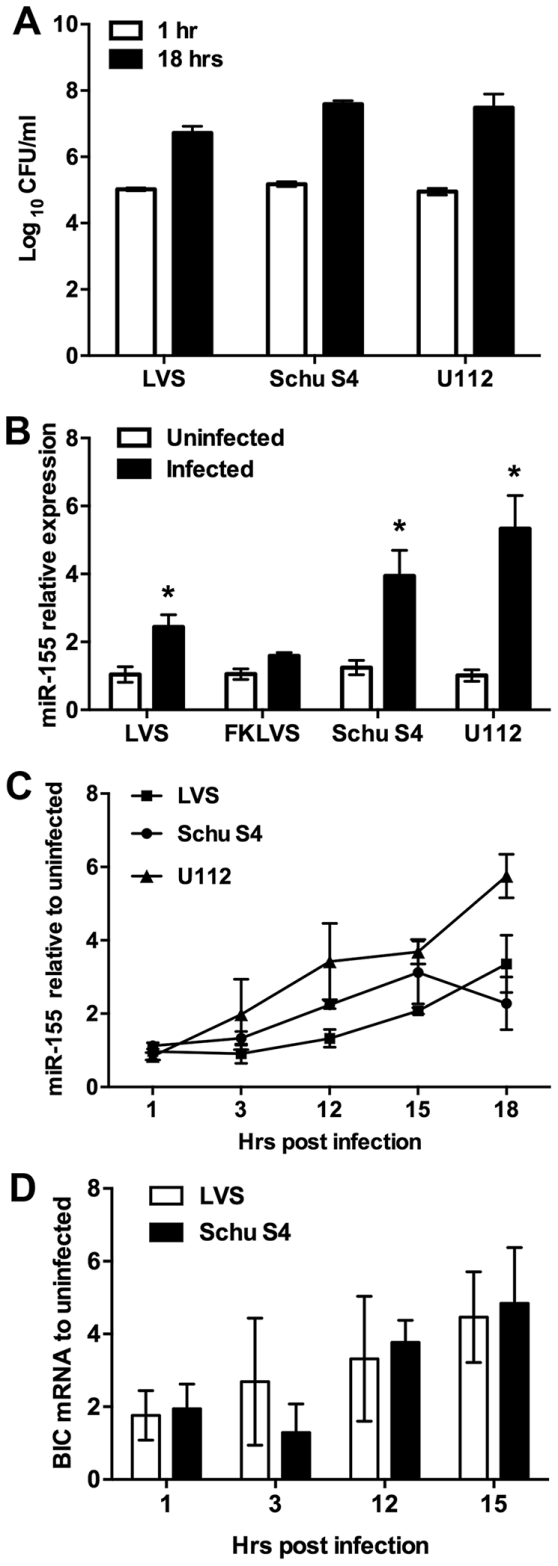
miR-155 is upregulated in MDMs infected with live *F. tularensis* or *F. novicida*. **A**. MDMs were infected with *F. tularensis* LVS, *F. tularensis* Schu S4 or *F. novicida* U112 at an MOI of 100∶1, and viable intracellular bacteria were quantified by measurement of CFU at 1 h and 18 h, as indicated. Data shown are the mean ±SEM from three independent experiments (n = 3). **B**. Relative miR-155 expression in control MDMs and cells that were infected for 18 h with live or formalin-killed (FK) LVS, or live U112 and Schu S4. Data are mean ±SEM (n = 3). *P<0.05 vs. the uninfected control. **C**. Time course of miR-155 induction in MDMs infected with LVS, Schu S4, or U112 were quantified by qRT-PCR. Data are the mean ±SEM (n = 3). **D**. Changes in *BIC* mRNA expression induced by LVS or Schu S4 were also quantified by qRT-PCR. Data are the mean ±SEM (n = 3). Data in panels B-D are normalized to the uninfected controls.

To address this issue, we directly compared the ability of *F. tularensis* LVS, *F. tularensis* Schu S4, and *F. novicida* U112 to induce miR-155 in MDMs. Macrophages were infected with each strain at an MOI of 100∶1, and CFU assays were performed at 1 and 18 h post-infection to quantify bacterial uptake and intracellular growth. As shown in Figures 2A–2B, all three bacterial strains replicated efficiently in MDMs over the time course examined, and miR-155 was significantly induced in each case, though not to the same extent. Specifically, miR-155 increased 2.44±0.36 fold in response to LVS, 3.94±0.74 fold in response to Schu S4, and 5.33±0.97 fold in response to U112 (Fig. 2B). In marked contrast, miR-155 induction was nearly ablated in MDMs infected with formalin-killed LVS, suggesting specificity for live bacteria.

Subsequent kinetic analyses indicated that miR-155 expression increased gradually 3–18 h after infection of MDMs with LVS or Schu S4 (Fig. 2C). A similar time course of miR-155 induction was observed in U112-infected MDMs (Fig. 2C). The gradual and delayed increase in miR-155 we report here contrasts sharply with the rapid and robust increase in miR-155 that occurs in macrophages exposed to LPS, other TLR ligands, or whole bacteria such as *Salmonella*, and *Listeria* [23], [24].

miR-155 is processed from exon 3 of the B cell Integration Cluster (*BIC* gene, recently renamed miR-155 host gene or *MIR155HG*), a region of human chromosome 21 that is expressed by activated B cells, T cells, monocytes and macrophages [37]. We therefore analyzed the time course of *BIC* mRNA induction by LVS and Schu S4, and show that, similar to miR-155, *BIC* transcripts increased gradually over the first 15 h after infection (Fig. 2D). Taken together, our data indicate that miR-155 is induced in MDMs by infection with viable *F. tularensis* as well as *F. novicida*, and further suggest that the rate and extent of miR-155 induction may differ significantly from other stimuli studied to date.

### miR-155 mediates suppression of SHIP-1 and MyD88 by translational repression

To define the role of miR-155 in *Francisella* infection, it is important to determine its downstream targets. Using bioinformatics analysis (TargetScan: http://www.targetscan.org) we identified miR-155 targets potentially relevant in *F. tularensis* infection, and focused on SHIP-1 and MyD88 as they are established true targets of this microRNA [38], [39]. SHIP-1, the Src homology 2 (SH2) domain containing inositol 5′ phosphatase, is a hematopoietic cell-specific enzyme that catalyzes conversion of PI(3,4,5)P_3_ into PI(3,4)P_2_, both of which are critical intermediates in the phosphatidylinositol 3- kinase (PI3K)/Akt signaling pathway [40]. As such, SHIP-1 is a candidate regulator of macrophage proinflammatory responses to *F. novicida* [41], but was reported not to be downregulated in cells infected with Schu S4 [29]. MyD88, Myeloid differentiation factor 88, is an adaptor protein that plays a central and critical role in both TLR and interleukin-1 receptor (IL-1R) signaling pathways [42]. For this reason, MyD88 is essential for innate defense against invading bacteria and other microbes, and as such contributes to control of primary infection of mice with both LVS and Schu S4 [43], [44].

**Figure 3:**
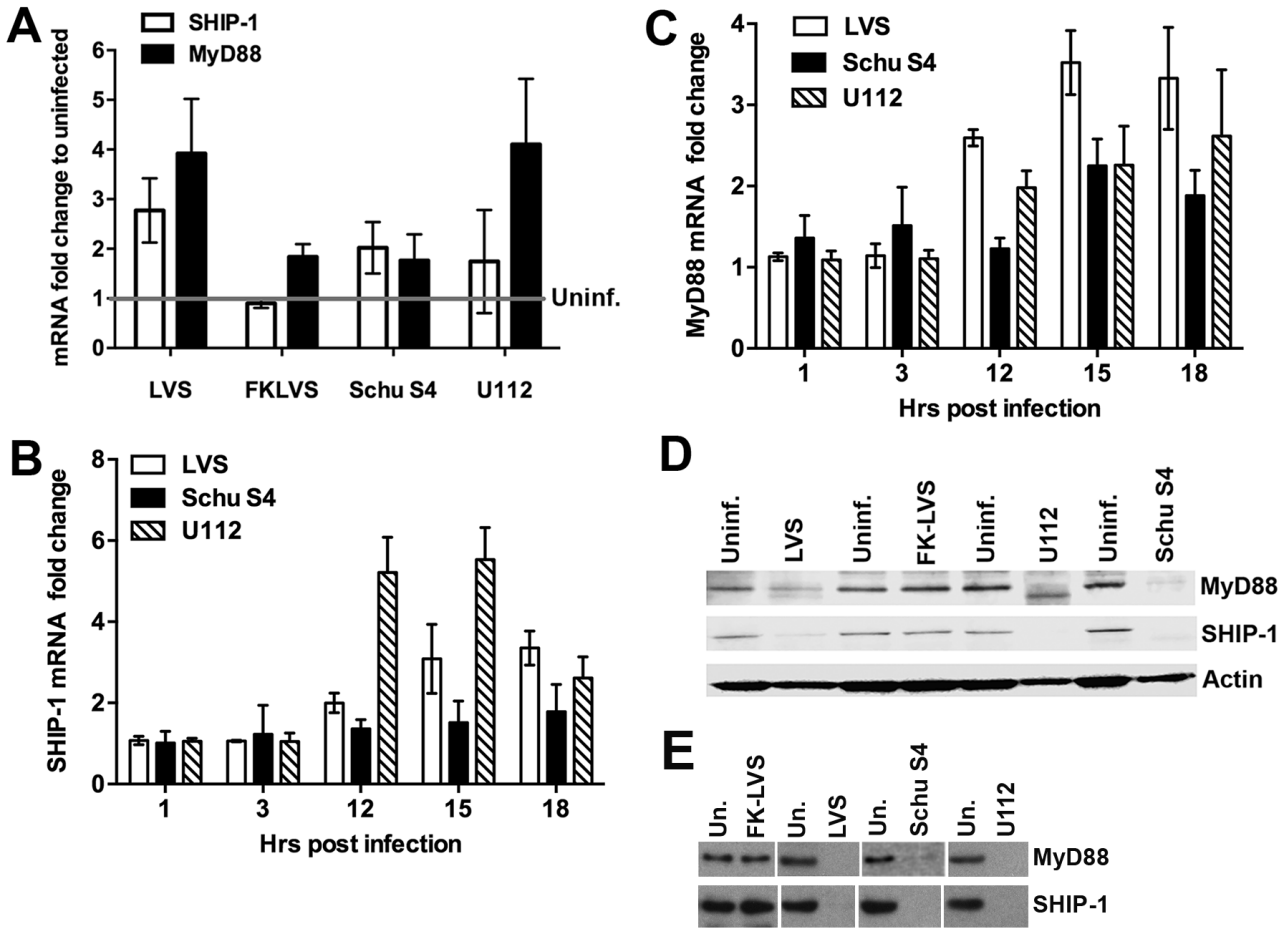
Post- translational depletion of MyD88 and SHIP-1 during *Francisella* infection. **A**. MDMs were infected for 18 h with live or formalin-killed (FK) LVS, live Schu S4, or live U112 as indicated, and MyD88 and SHIP-1 mRNAs were measured by qRT-PCR with GAPDH as the internal control. Data shown are the mean ±SEM (n = 3), and were normalized to the uninfected control. **B–C**. SHIP-1 (C) and MyD88 (D) mRNAs were upregulated in MDMs infected with LVS, Schu S4 or U112. Data shown are the mean ±SEM (n = 3). **D**. MDMs were left untreated or were infected with live or formalin-killed LVS, live Schu S4 or live U112 for 18 h. Immunoblots of MDM lysates demonstrate disappearance of MyD88 and SHIP-1 protein following infection with live *Francisella*. Actin was used as the loading control. **E**. Immunoblots from additional experiments exposed for longer periods of time confirm depletion of MyD88 and SHIP-1. Data in panels D and E are representative of at least three independent determinations.

microRNAs inhibit protein synthesis by inducing mRNA degradation or by translational repression [45]. We therefore quantified SHIP-1 and MyD88 mRNA and protein in control and *Francisella*-infected MDMs. As judged by qRT-PCR analysis, SHIP-1 and MyD88 transcripts were not downregulated in MDMs infected with LVS, Schu S4 or U112. Rather, the abundance of both target mRNAs increased 2–4 fold over baseline by 18 h post-infection (Fig. 3A). Additional experiments showed that SHIP-1 (Fig. 3B) and MyD88 (Fig. 3C) mRNAs were relatively unchanged for the first few hours after infection with LVS, Schu S4, or U112 and then increased between 12 and 18 h, the latest time point examined. On the other hand, infected MDMs were depleted of SHIP-1 and MyD88 protein as indicated by immunoblotting (Figs. 3D and 3E). Moreover, disappearance of the target proteins was apparent by 12 h of infection with LVS (Fig. S1B) in agreement with the time course of miR-155 induction. Consistent with its inability to induce miR-155, infection of MDMs with killed LVS had little or no effect on SHIP-1 and MyD88 mRNAs (Fig. 3A) and did not confer protein depletion (Figs. 3D and 3E). These data strongly suggest that miR-155 acts at the translational level to deplete *Francisella*-infected MDMs of both MyD88 and SHIP-1.

**Figure 4:**
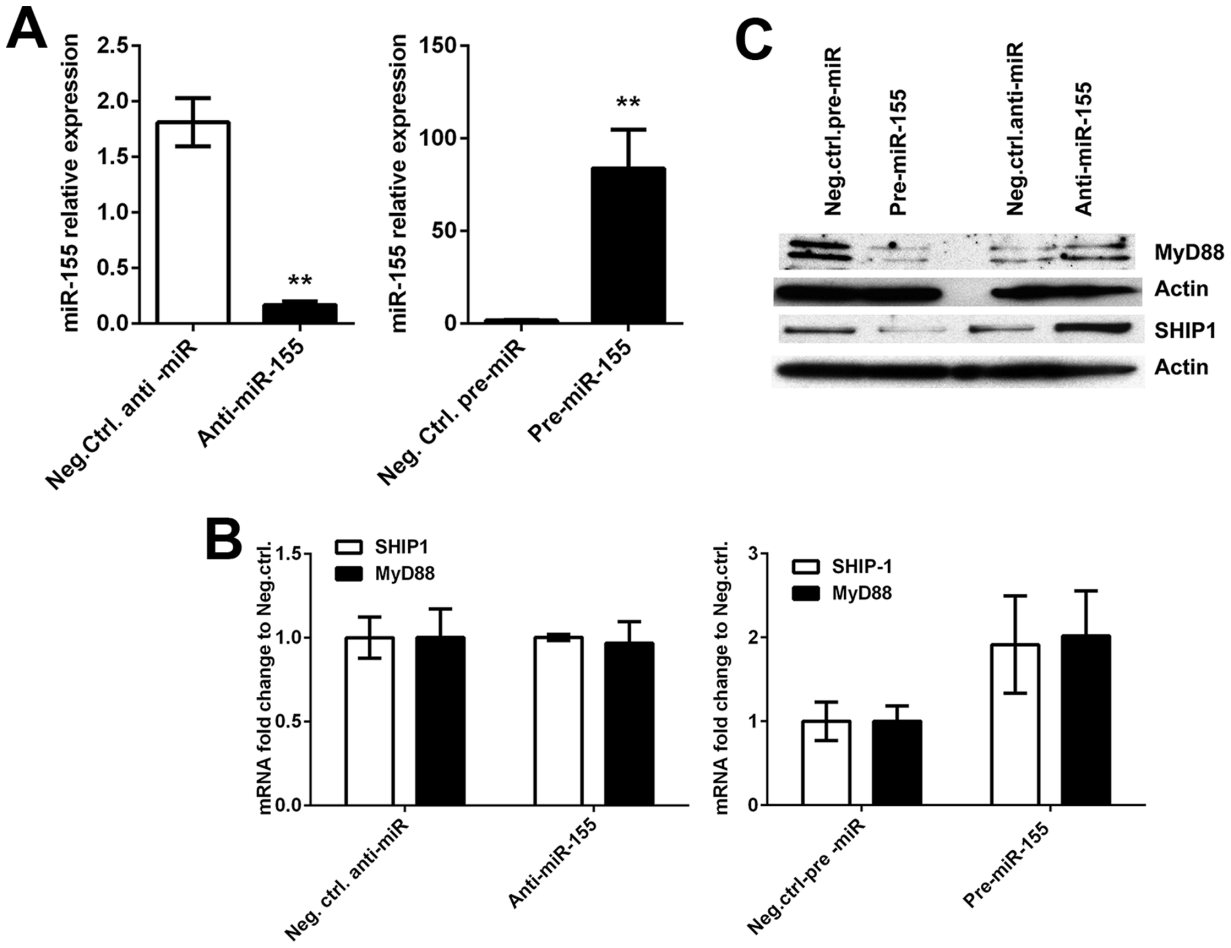
Direct manipulation of miR-155 levels alters MyD88 and SHIP-1 protein abundance, but not their mRNAs. MDMs were transfected with negative control and miR-155 mimic (pre-miR), or with negative control and miR-155 inhibitor (anti-miR) as indicated. **A**. miR-155 expression was analyzed using a Taqman microRNA assay and was normalized to RNU48. Data are mean ±SEM (n = 3). **P<0.01 vs. the negative control. **B**. SHIP-1 and MyD88 mRNAs were quantified 48 h after transfection using qRT-PCR with GAPDH as the internal control. Data are mean ±SEM (n = 3). **C**. SHIP-1 and MyD88 proteins were detected in MDM lysates by immunoblotting. β-actin was used as the loading control. Data shown are from one experiment representative of three.

To confirm the role of miR-155 in target protein downregulation, we determined whether overexpression or inhibition of miR-155 would lead to significant changes in SHIP-1 and MyD88 abundance. To this end, MDMs were transfected with pre-miR-155 or anti-miR-155 as well as negative control pre-miRs or anti-miRs using reagents from Ambion and Dharmacon, according to the manufacturer's recommendations. Transfection efficiency was >60% for all oligonucleotides used as determined microscopically by Cy3 labeled siRNA transfection (data not shown). microRNA overexpression or inhibition was not cytotoxic as measured by lactate dehydrogenase release (Fig. S2A). However, preliminary studies suggest that miR-155 overexpression favored the viability of MDMs treated with the PI3K inhibitor LY294002 (Fig. S2B). RNA was extracted 48 h post-transfection, and miR-155 expression was analyzed by qRT-PCR. Overexpression and inhibition of miR-155 markedly increased and decreased mature miR-155 expression levels compared to negative control-transfected cells (Figs. 4A), respectively. Despite the profound changes in miR-155 abundance, miR-155 overexpression or inhibition had little or no effect on SHIP-1 or MyD88 mRNA levels (Fig. 4B). In contrast, SHIP-1 and MyD88 protein levels were decreased by miR-155 overexpression, whereas miR-155 antagonists had the opposite effect (Fig. 4C). Collectively, our data support a model whereby miR-155 regulates SHIP-1 and MyD88 abundance in MDMs during *Francisella* infection.

### miR-155 does not attenuate bacterial uptake or replication in MDMs

**Figure 5:**
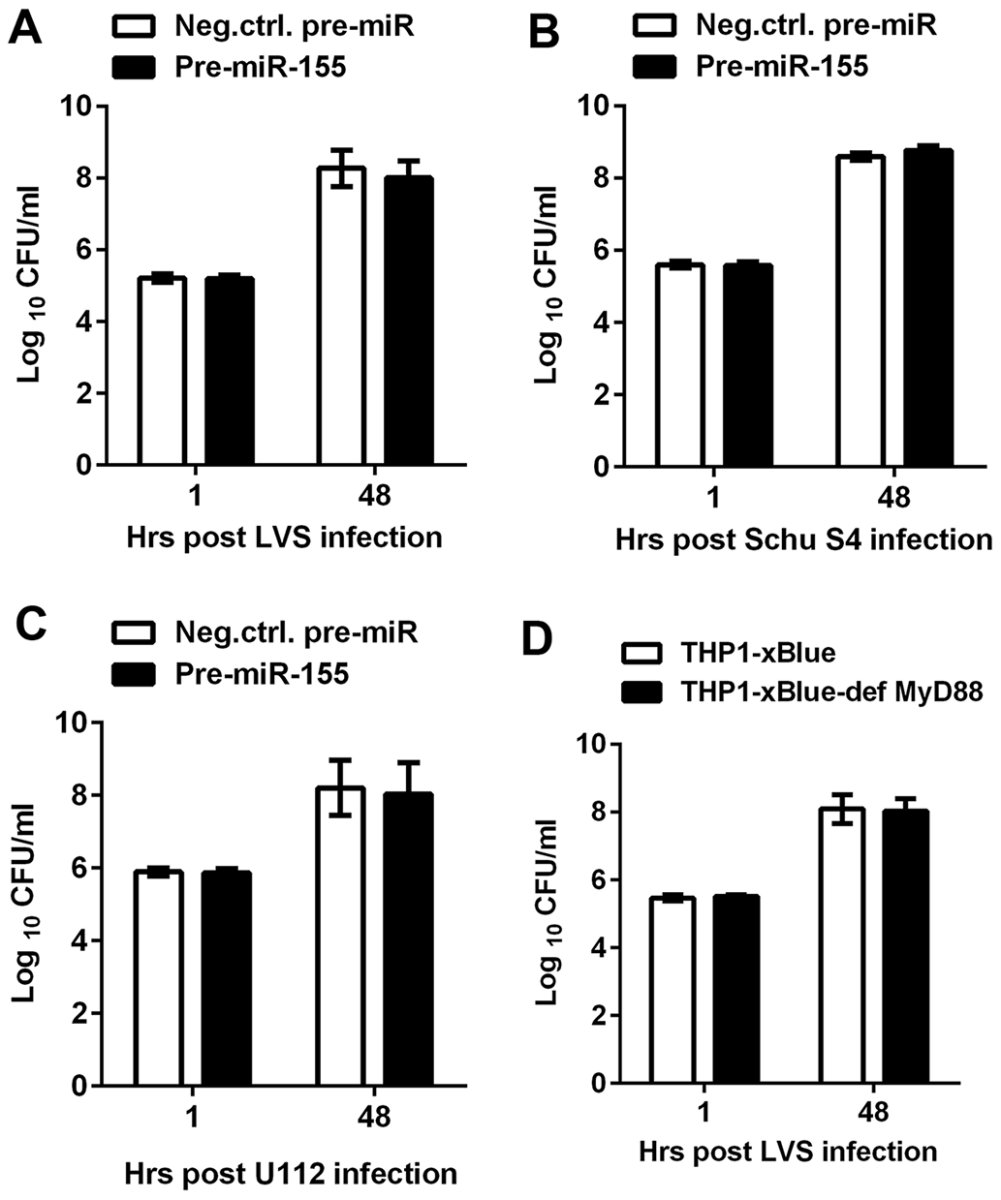
Neither miR-155 overexpression nor MyD88 deficiency alters *F. tularensis* replication in human macrophages. **A–C**. MDMs were infected with LVS (A), Schu S4 (B), or U112 (C) 48 h after transfection with miR-155-specific or negative control pre-miRs, and viable intracellular bacteria were quantified at the indicated time points by measurement of CFU. Data shown are the mean ±SEM (n = 3). **D**. LVS replicates normally in control (xBlue) and MyD88-deficient (x-Blue-def MyD88) THP-1 macrophages. Data are the mean ±SEM (n = 3).

To address whether enhanced miR-155 expression was deleterious to bacterial growth in MDMs, we investigated the bacterial burden in our gain- or loss-of-function of microRNA experiments. Forty-eight hours after transfection, cells were infected with LVS or Schu S4 at an MOI of 100∶1, and CFU assays were performed at 1 and 48 h post-infection. Significant overexpression of miR-155 did not change bacterial uptake by MDMs compared to the negative control, and had no apparent effect on LVS (Fig. 5A), Schu S4 (Fig. 5B) or U112 (Fig. 5C) intramacrophage growth. Furthermore, significant inhibition of miR-155 also failed to show any effect on bacterial uptake or growth as compared with the anti-miR-control (data not shown). We next used MyD88-deficient THP-1 monocytes (THP-1-xBlue-def-MyD88 cells) (InvivoGen) to assess directly whether the absence of this miR-155 target was advantageous to the uptake or growth of the bacteria. In our hands, LVS infection was not affected by MyD88 deficiency as compared to Thp-1-xBlue cell controls (Fig. 5D). However, LVS did not induce miR-155 expression in THP-1-xBlue-def-MyD88 cells (data not shown), in keeping with the fact that induction of this microRNA requires TLR signaling [35].

### miR-155 and TNFα are induced with similar kinetics

**Figure 6:**
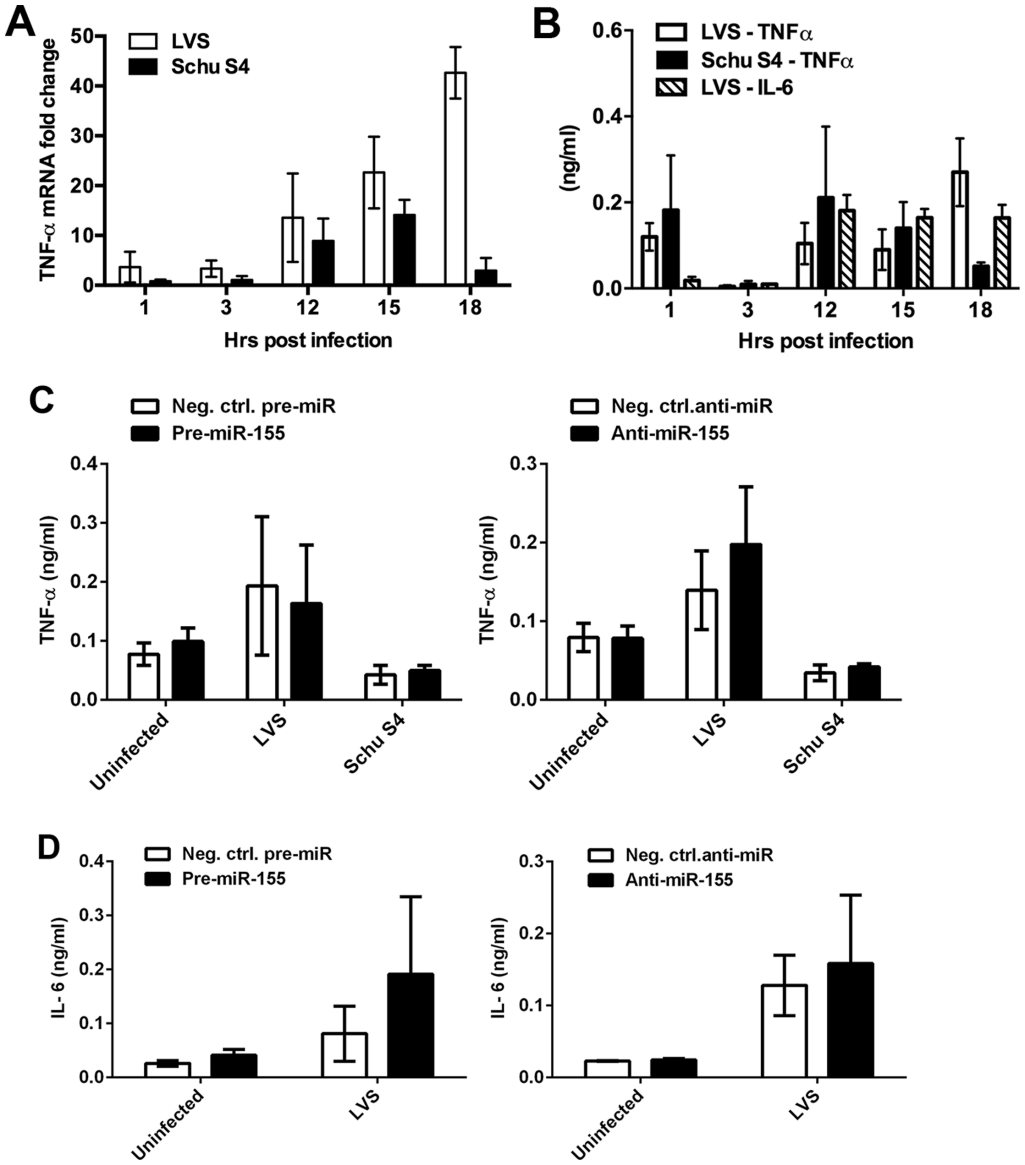
Manipulation of miR-155 does not significantly alter TNFα or IL-6 secretion triggered by *F. tularensis* infection. **A**. Effects of LVS and Schu S4 on TNFα mRNA expression were analyzed by qRT-PCR. Data shown are the mean ±SEM (n = 3) and were normalized to GAPDH. **B**. MDMs were infected with LVS or Schu S4, and secreted TNFα and IL-6 were quantified at the indicated time points by ELISA. Data shown are the mean ±SEM (n = 3). **C–D**. MDMs were transfected with the indicated pre-miRs and anti-miRs 48 h prior to infection with LVS or Schu S4, as indicated. At 24 h post-infection the amount of TNFα (C) and IL-6 (D) secreted into the extracellular medium was quantified by ELISA. Data shown are the mean ±SEM (n = 3).

Macrophages are central players in the regulation of systemic bacterial infection-induced immune responses, which includes the synthesis and secretion of cytokines and interleukins for intercellular communication. We show here that *Francisella*-induced miR-155 caused depletion of MyD88 and SHIP-1 in MDMs, and it is established that the proinflammatory cytokine response evoked by this organism is low in magnitude compared to classical stimuli [46], [47], but whether these processes are mechanistically linked is unclear. The host response to *F. tularensis* is driven by TLR2 signaling initiated from phagosomes at the earliest stages of infection [48], and the TLR-MyD88-NF-κB pathway is also critical for miR-155 induction [35]. This shared dependence on TLR signaling suggested that miR-155 and proinflammatory cytokines might be upregulated in parallel. To test this, we quantified the rate and extent of TNFα mRNA induction in the same samples that were used to quantify miR-155 in Figure 2C, and also measured TNFα and IL-6 secretion by ELISA. Our results demonstrate that the kinetics of TNFα induction in response to LVS or Schu S4, like induction of miR-155 (Fig. 2C), was very low during the first 3 h after infection and then increased between 12 and 18 h (Fig. 6A), whereas the ELISA data confirm that *F. tularensis*-evoked cytokine secretion was very low (Fig. 6B). Indeed, IL-6 was not consistently detected following Schu S4 infection (data not shown). On the other hand, TNFα mRNA increased 200-fold after infection with U112 (Fig. S3A), and TNFα and IL-6 levels in the culture medium (Fig. S3B) were up to 30-fold higher than we observed for LVS and Schu S4, confirming that *F. novicida* is significantly more proinflammatory than *F. tularensis* [49].

### miR-155 is not sufficient to modulate TNFα and IL-6 secretion triggered by *F. tularensis*

As miR-155 induction and MyD88 depletion occurred in parallel with TNFα expression in MDMs, and were therefore too slow to prevent cytokine production triggered by bacterial uptake, we used an alternative strategy to assess the functional consequences of miR-155 induction. Specifically, we tested whether miR-155 overexpression or inhibition prior to infection could alter the magnitude of *F. tularensis*-triggered TNFα or IL-6 secretion. In our hands, overproduction or inhibition of miR-155 in MDMs did not significantly alter TNFα mRNA levels (not shown) or TNFα secretion initiated by LVS or Schu S4 infection (Fig. 6C). IL-6 was also unchanged upon LVS infection of MDMs in which miR-155 was overexpressed or downregulated (Fig. 6D), and was not detected following infection with Schu S4 (data not shown). These results demonstrate that profound overexpression of miR-155 did not enhance proinflammatory cytokine secretion during infection with LVS or Schu S4, which contrasts markedly with the ability of this microRNA to enhance proinflammatory responses during *F. novicida* infection of human monocytes or murine macrophages [29]. Rather, the miR-155-dependent downregulation of MyD88 shown in Figure 4 strongly suggests that this microRNA plays an anti-inflammatory role in MDMs. At the same time, our data also show that upregulation of key TLR pathway intermediates upon miR-155 inhibition (Fig. 4C) was not sufficient to override the low proinflammatory nature of *F. tularensis* (Fig. 6C–D), which is believed to be driven by a paucity of TLR-activating ligands and the shielding effects of surface O-antigen and capsule [4], [47].

### LVS infection and miR-155 overexpression inhibit LPS-stimulated TNFα secretion

**Figure 7:**
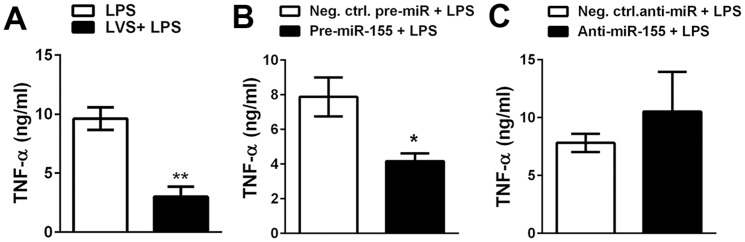
LVS infection and miR-155 overexpression impair LPS-stimulated TNFα secretion. **A**. Control MDMs or cells that had been infected with LVS for 18 h were stimulated with 100 ng/ml *E. coli* LPS, and after 6 h at 37°C the amount of TNFα in the supernatant was quantified by ELISA. Data shown are the mean ±SEM (n = 3). **B–C**. MDMs were transfected with the indicated pre-miRs and anti-miRs 48 h prior to stimulation with *E. coli* LPS, and TNFα secretion was quantified as for panel A. Data are the mean ±SEM (n = 3).

A growing body of data indicates that the ability of *F. tularensis*-infected macrophages, neutrophils and dendritic cells to be activated by heterologous stimuli is profoundly impaired [6], [11], [12], [50]. To further explore the function of miR-155 in MDMs, we infected cells with LVS for 18 h (to allow downregulation of MyD88 and SHIP-1), and then stimulated the cells with 100 ng/ml *E. coli* LPS for 6 h, and quantified TNFα secretion (Fig. 7A). Our data demonstrate that LVS infection markedly impaired LPS-stimulated TNFα secretion in MDMs. We also demonstrate a direct role for miR-155 in this process, as overexpression of miR-155 was sufficient to significantly inhibit TNFα secretion in the absence of LVS infection (Fig. 7B), whereas cytokine secretion was enhanced by miR-155 downregulation, although this did not reach statistical significance (Fig. 7C). Collectively, these data demonstrate the ability of miR-155 to modulate cytokine production, and provide direct evidence of its anti-inflammatory function in MDMs.

## Discussion

In this study we undertook what is, to our knowledge, the first overall analysis of the human macrophage microRNA response to *F. tularensis*, and demonstrate that a small subset of microRNAs were significantly differentially regulated in MDMs within 18 hours of infection. Of these, miR-155 was investigated further as several targets of this microRNA have been identified, and it has been shown to have both pro- and anti-inflammatory effects that are likely context-specific. In keeping with this, we identified shared and distinct effects of *F. tularensis* on miR-155 as compared with other stimuli characterized to date, and we propose, as discussed below, that downregulation of MyD88 contributes to active inhibition of the inflammatory response during tularemia, defined as impaired responsiveness to secondary stimuli [12], [51]. As such, the results of this study are consistent with the ability of miR-155 to act as a negative feedback regulator of the inflammatory response [22].

The central finding of this study is our demonstration that *F. tularensis* infection of MDMs leads to downregulation of MyD88 and SHIP-1 via a miR-155-dependent mechanism. miR-155 induction by LPS and other TLR agonists, including Gram-negative and Gram-positive bacteria, has been studied in detail and is consistently described as a rapid and sustained response. Increased expression of miR-155 is generally apparent by ∼90 minutes, peaks within 3-6 hours, and can be maintained at a high level for as long as 24–48 hours [23], [24], [35], [52]–[54]. At the same time, the magnitude of the response appears to be cell type-specific, with robust induction (∼50-500-fold) occurring in primary murine bone marrow-derived macrophages, RAW264.7 cells, and human monocytes, whereas moderate induction (less than 5-fold) is observed in primary human MDMs and THP-1 cells [55]–[57]. This pattern extends to other microRNAs besides miR-155 and has been attributed to a significant increase in basal microRNA levels [57], resulting from an increase in Dicer and other proteins involved in microRNA biogenesis during monocyte to macrophage differentiation [58]. Thus, the magnitude of the human macrophage microRNA response to *F. tularensis* that we report here (∼2-4-fold) is consistent with previous studies of this cell type. Our data also indicate that depletion of miR-155 targets was achieved at the protein level rather than mRNA degradation, indicating translational repression, and this same mechanism is known to mediate miR-155-mediated MyD88 downregulation during *Helicobacter pylori* infection [39].

On the other hand, the kinetics of miR-155 induction in our system was slow and delayed, and as such diverged from the established paradigm. What accounts for this is unclear, but the time course of miR-155 upregulation we report is concordant with the kinetics of *BIC/miR155HG* expression, as well as the time course of target protein depletion in *F. tularensis*-infected cells. As an additional control we treated MDMs with *E. coli* LPS, and as expected miR-155 levels were elevated within 2 hours, and peaked at 6 hours (data not shown). Although elucidation of the underlying molecular events is beyond the scope of this study, it is conceivable that signaling from cytosolic *F. tularensis* might contribute to miR-155 induction, particularly since *F. tularensis* replication in the cytosol of macrophages ensues by 6 hours after infection, and because the response to killed LVS, which are trapped inside phagosomes, was impaired. A possible role for cytosolic signaling is not without precedent as NOD1/2 sensing of peptidoglycan can upregulate miR-155 during infection with other organisms [59].

In the absence of MyD88 macrophages are refractory to stimulation by most TLR agonists as well as IL-1β. Our data strongly suggest that miR-155-dependent downregulation of this adapter ∼18 hours after infection plays a significant and previously unappreciated role in active inhibition of the inflammatory response by *F. tularensis* (Fig. 7). Consistent with this, studies of human subjects performed in the 1960s demonstrate that acute infection with Schu S4 inhibits responses to subsequently administered *Salmonella typhi* endotoxin by 60% [51]. Nonetheless, Telepnev *et al*., demonstrated that the ability of LVS-infected macrophages to secrete TNFα after stimulation with *E. coli* LPS or bacterial lipopeptides is impaired within 5 hours via a mechanism that correlates with MAP kinase (MAPK) inhibition [11], [60]. Additional studies suggest that this is mediated by MAPK phosphatase-1 (MKP-1), which is transiently expressed 1 hour after infection with LVS or Schu S4 [28], [61]. Specifically, signaling at forming phagosomes triggers MyD88- and PI3K-dependent upregulation of MKP-1, which plays an established role in negative feedback control of proinflammatory cytokine production by deactivating MAPKs [62]. All our experiments utilized bacteria opsonized with normal human serum, and ligation of CR3 by opsonized *F. tularensis* provides an additional signal leading to PI3K-dependent MKP-1 activation [28]. Considered together, the data support a model in which MKP-1 confers early attenuation of proinflammatory cytokine production that is reinforced at later stages of infection by miR-155-dependent downregulation of MyD88. In this regard it is of interest that MKP-1-deficient macrophages express higher levels of miR-155 than wild-type cells [63].

**Figure 8:**
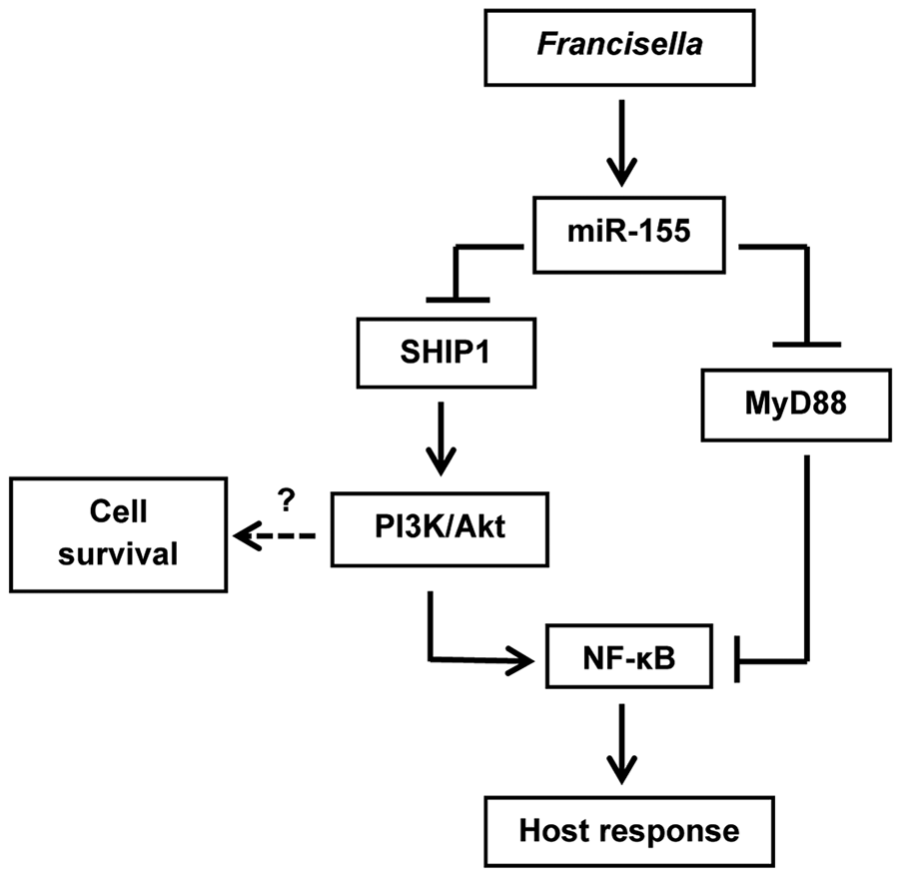
Model of miR-155-mediated regulation of the inflammatory response during *F. tularensis* infection. *F. tularensis* lipoproteins trigger TLR2 signaling in macrophages [4], which results in the MyD88-dependent upregulation of miR-155 by 18 h post-infection (Fig. 2). This microRNA is part of a negative feedback loop that confers downregulation of MyD88 and SHIP-1 by translational repression (Fig. 3). Loss of MyD88 impairs further TLR2 and IL-1 receptor signaling, and as such curtails inflammation, including TNFα secretion (Fig. 7). In contrast, depletion of SHIP-1 enhances signaling through the PI3K/Akt pathway [14], [38], [67], which is critical for macrophage survival as well as chemotaxis, macropinocytosis and phagocytosis [68]–[71] (Fig. S2B). We therefore propose that downregulation of MyD88 impairs host defense, whereas depletion of SHIP-1 may favor MDM survival and bacterial dissemination during tularemia.

At least two of the other microRNAs identified in our screen also inhibit macrophage proinflammatory capacity. miR-150 also targets MyD88 [64], whereas miR-146 targets IL-1 receptor-associated kinase 1 (IRAK1), IRAK2, and TNF receptor-associated factor 6 (TRAF6), key adaptor molecules downstream of TLRs and cytokine receptors [53], [56], [65]. A growing body of data implicates miR-146 and miR-155 in macrophage tolerance to TLR2 agonists as well as LPS [53], [54], [56], [65], and it will therefore be of interest in future studies to determine if miR-150, miR-146, and miR-155 synergize to inhibit macrophage activation capacity during tularemia. At the same time, miR-155 was originally defined as an onco-miR as its expression is enhanced in many cancers [66]. Thus, downregulation of SHIP-1 enhances basal and stimulated class I PI3K signaling that is essential for macrophage survival [14], [38], [67]. In agreement with this, our preliminary data show that miR-155 overexpression may diminish death of MDMs treated with PI3K inhibitors (Fig. S2B). As PI3Ks are also required for macrophage adhesion, chemotaxis, and phagocytosis [68]–[71], the data suggest a working model in which downregulation of MyD88 and SHIP-1 act in concert to impair host defense, yet sustain macrophage viability and other aspects of cell function required for *F. tularensis* growth and dissemination (Fig. 8). Nonetheless, this scenario is likely incomplete as other miR-155 targets such as Fas-associated death domain protein (FADD), IκB kinase ε (IKKε), the receptor interacting serine-threonine kinase 1 (RIPK1), transcription factor PU.1, and CCAAT enhancer binding protein β (C/EBPβ), that were not examined here, can also influence macrophage survival [54], [72], [73]. Furthermore, among the other microRNAs identified in our screen, miR-886 [74], [75] is also associated with cell proliferation, survival, and cancer, providing additional indirect support for the hypothesis that *F. tularensis* modulates macrophage survival.

In addition to *F. tularensis*, *H. pylori*, *Listeria monocytogenes*, *Mycobacterium tuberculosis,* and *Salmonella enterica* serovar Typhimurium have all been shown to manipulate microRNA expression in host cells [20]. In this regard it is noteworthy that miR-155 is upregulated by all these pathogens whereas other microRNAs show partial overlap or appear to be specific for a single organism [20](and this study). Similar to our findings (Fig. 5), miR-dependent manipulation of MyD88 does not affect phagocytosis or intracellular growth of *L. monocytogenes in vitro* [24]. However, effects of miR-155 on B cells, T cells, and dendritic cells [76], [77] are not represented in our *in vitro* assay system, and merit further analysis given the critical roles of T cells and TLR2/MyD88 in defense against *F. tularensis in vivo* [43], [44], [78]. Similarly, null mutations in MyD88 significantly curtail host defense in mice, yet have no apparent effect on phagocytosis or bacterial survival in macrophages *in vitro* [43], [79].

In keeping with its ability to have proinflammatory as well as anti-inflammatory effects, miR-155 can be induced by TNFα via autocrine and paracrine signaling [35]. TNFα mRNA accumulated in *F. tularensis*-infected MDMs (Fig. 6A), in agreement with the ability of this microRNA to increase transcript half-life y acting at the 3 prime UTR of TNFα mRNA to release self-inhibitory effects [54] or increase mRNA stability [80]. However, proinflammatory cytokine secretion by *F. tularensis*-infected cells was very low (Fig. 6B), and when tested directly this amount of recombinant TNFαwas not sufficient to induce miR-155 in MDMs (our unpublished data). In addition, overexpression or inhibition of miR-155 did not significantly alter basal or infection-induced TNFαsecretion (Fig. 6C). To our knowledge, post-transcriptional inhibition of cytokine secretion during tularemia has not been reported previously. miR-125b, which directly targets TNFα, is downregulated by LPS and upregulated by *M. tuberculosis* [20]. However, neither miR-125b nor other microRNAs known to target TNFα (miR-221 and miR-579) [53] were detected in our screen.

*F. novicida* is sigificantly more proinflammatory than *F. tularensis* LVS or Schu S4 [28] (and compare Fig. S3 to Figs. 6A–6B). Previously, Cremer *et al*. [29] demonstrated that miR-155 is more strongly upregulated by *F. novicida* than *F. tularensis* Schu S4 in human monocytes, leading to downregulation of SHIP-1 in response to the former, but not to the latter organism. Moreover, ectopic expression of miR-155 significantly enhanced *F. novicida*-triggered proinflammatory cytokine secretion [29]. On the other hand, we find that miR-155 was induced in MDMs in response to all three bacterial strains tested (Figs. 2B–C), and although the magnitude of the induction differed to some extent, SHIP-1 and MyD88 were downregulated in cells infected with *F. tularensis* LVS or Schu S4, as well as *F. novicida* (Fig. 3D). In addition, downregulation of SHIP-1 by *F. tularensis* infection, miR-155 overexpression, or miR-155 overexpression followed by infection with LVS or Schu S4 was not proinflammatory in MDMs, and cytokine secretion was not enhanced (Fig. 6C–D). It is likely that multiple factors contribute to the divergent findings from these two studies, including use of different mononuclear phagocyte types, differences in bacterial growth media, and the presence or absence of serum opsonins. For example, we show that MyD88 transcripts accumulated in MDMs despite dissappearance of the cognate protein. Conversely, MyD88 mRNA is downregulated in human monocytes infected with Schu S4 or U112 [81], but whether this is linked to miR-155 is not yet known. We also used bacteria grown in brain heart infusion broth, which for us and others confers a host-adapted phenotype that results in maximum inhibiton of innate defense mechanisms [46], [47]. CR3-mediated immune suppression is relatively specific for Schu S4 and has little to no effect on *F. novicida* [28], and we used opsonized bacteria whereas Cremer *et al*. did not. Thus, it is possible that additional or redundant mechanisms of inhibition evoked by *F. tularensis*, such as MKP-1 activation [28], [61], are absent in *F. novicida*. Consistent with this, TNFαand IL-6 secretion by *F. tularensis*-infected MDMs declined over the first 3 hours of infection (Fig. 6B), whereas cytokine secretion by *F. novicida*-infected MDMs increased at least 30-fold over this same time period (Fig. S3B). Distinct effects of *F. novicida* on other miR-155 targets or other microRNAs must also be considered.

In summary, the results of this study identified microRNAs that were significantly differentially regulated by *F. tularensis* infection of primary human macrophages. In particular, we demonstrate that miR-155 was induced, and show that this led to downregulation of MyD88 and SHIP-1 via translational repression. At the same, the kinetics of miR-155 induction and target depletion in our system were slow, and did not contribute to the weak cytokine response triggered by uptake of this pathogen. Rather, the negative regulatory role of miR-155 was apparent at a later stage of infection and was instrumental in the ability of infected MDM to resist activation by *E. coli* LPS. As our understanding of the effects of microRNAs on host defense and cell survival is rapidly evolving, it will be of interest in future studies to elucidate the possible effects of other miR-155 targets and the other microRNAs we identified in tularemia pathogenesis *in vivo* and *in vitro*, and to determine if miR-155 undermines the efficacy of therapeutic strategies that attempt to control *F. tularensis* by activation of infected macrophages.

## Supporting Information

Figure S1 ***Francisella*** **replicates efficiently in MDMs and induces depletion of MyD88 and SHIP-1.** MDMs were infected at an MOI of 100∶1 with *F. tularensis* strains LVS and Schu S4, or *F. novicida* strain U112 for 18 h (A) or 12 h and 18 h (B). **A**. Low magnification (*top row*) and high magnification (*bottom row*) confocal images show bacteria in green and lamp-1 in red and are representative of more than three independent experiments. **B**. Immunoblots of MDM lysates demonstrate LVS-induced downregulation of MyD88 and SHIP-1 at 12 h and 18 h post-infection. Actin was used as a loading control. Data shown are representative of three independent experiments.
(TIF)

Figure S2 **Effects of miR-155 on MDM viability.** **A**. Modulation of miR-155 abundance is not directly toxic. MDMs were left untreated, were exposed to buffer alone, or were transfected with control or miR-155 pre-miR and anti-miR as indicated. Cytotoxicity was measured after 48 h and indicates the amount of lactate dehydrogenase released into the cell supernatant as compared with the positive control (MDMs lysed with 9% Triton X-100 in water). Data are the mean ±SEM (n = 3). ****p<0.0001 vs. all other samples. **B**. Over-expression of miR-155 induced by transfection with pre-miR-155 constructs partially protects MDMs from death induced by treatment with the PI3K inhibitor LY294002 (40 µM, 48 h). Cell death was assessed by propidium iodide staining and flow cytometry. Control MDMs were transfected with negative control pre-miR constructs. Data shown are from one preliminary experiment.
(TIF)

Figure S3 ***F. novicida*** **induces secretion of proinflammatory cytokines.** **A–B**. MDMs were infected with *F. novicida* strain U112 at an MOI of 100∶1. At the indicated time points, TNFα mRNA upregulation was quantified by qRT-PCR (A) and secretion of TNFα and IL-6 into the extracellular medium was quantified by ELISA (B). Data shown in each graph are the mean ±SEM of three independent experiments.
(TIF)
