# Supplementary figures and images for: Differential Expression of microRNAs in Francisella tularensis-Infected Human Macrophages: miR-155-Dependent Downregulation of MyD88 Inhibits the Inflammatory Response
Source: PLoS One. 2014 Oct 8;9(10):e109525. doi: 10.1371/journal.pone.0109525 (PMC4190180; doi:10.1371/journal.pone.0109525)

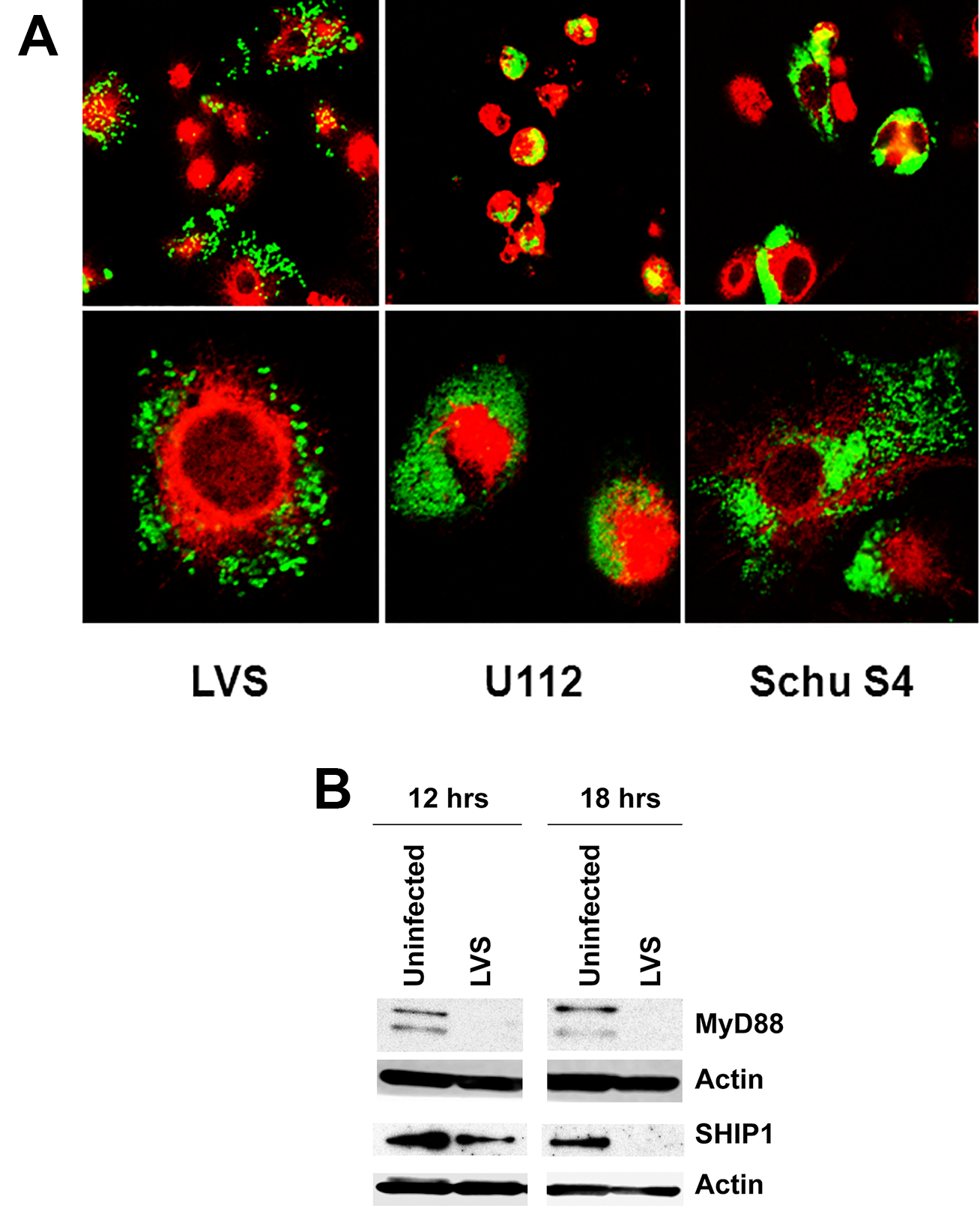

Supplement: Figure S1 — Francisella replicates efficiently in MDMs and induces depletion of MyD88 and SHIP-1. MDMs were infected at an MOI of 100∶1 with F. tularensis strains LVS and Schu S4, or F. novicida strain U112 for 18 h (A) or 12 h and 18 h (B). A. Low magnification (top row) and high magnification (bottom row) confocal images show bacteria in green and lamp-1 in red and are representative of more than three independent experiments. B. Immunoblots of MDM lysates demonstrate LVS-induced downregulation of MyD88 and SHIP-1 at 12 h and 18 h post-infection. Actin was used as a loading control. Data shown are representative of three independent experiments. (TIF) [file pone.0109525.s001.tif]

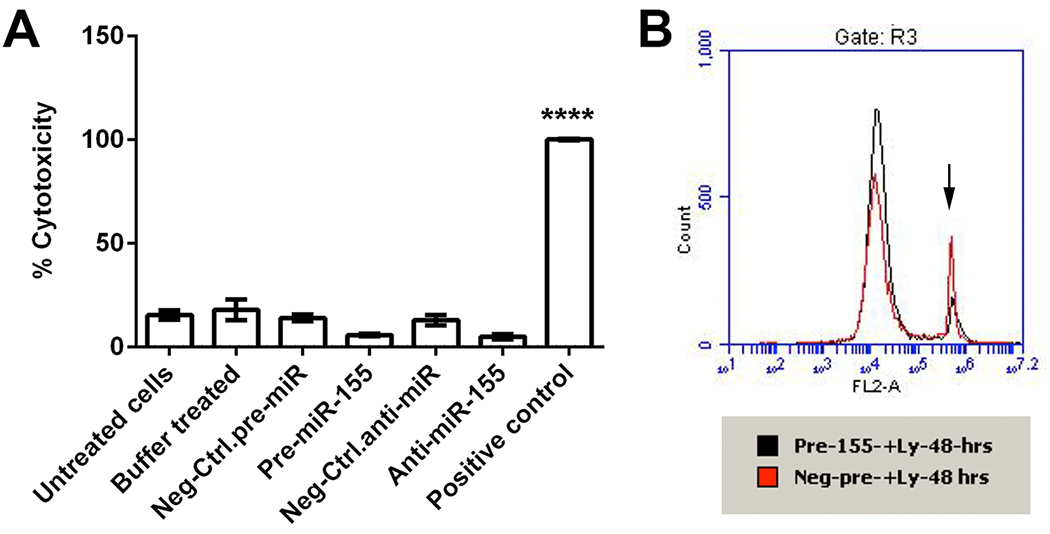

Supplement: Figure S2 — Effects of miR-155 on MDM viability. A. Modulation of miR-155 abundance is not directly toxic. MDMs were left untreated, were exposed to buffer alone, or were transfected with control or miR-155 pre-miR and anti-miR as indicated. Cytotoxicity was measured after 48 h and indicates the amount of lactate dehydrogenase released into the cell supernatant as compared with the positive control (MDMs lysed with 9% Triton X-100 in water). Data are the mean ±SEM (n = 3). ****p<0.0001 vs. all other samples. B. Over-expression of miR-155 induced by transfection with pre-miR-155 constructs partially protects MDMs from death induced by treatment with the PI3K inhibitor LY294002 (40 µM, 48 h). Cell death was assessed by propidium iodide staining and flow cytometry. Control MDMs were transfected with negative control pre-miR constructs. Data shown are from one preliminary experiment. (TIF) [file pone.0109525.s002.tif]

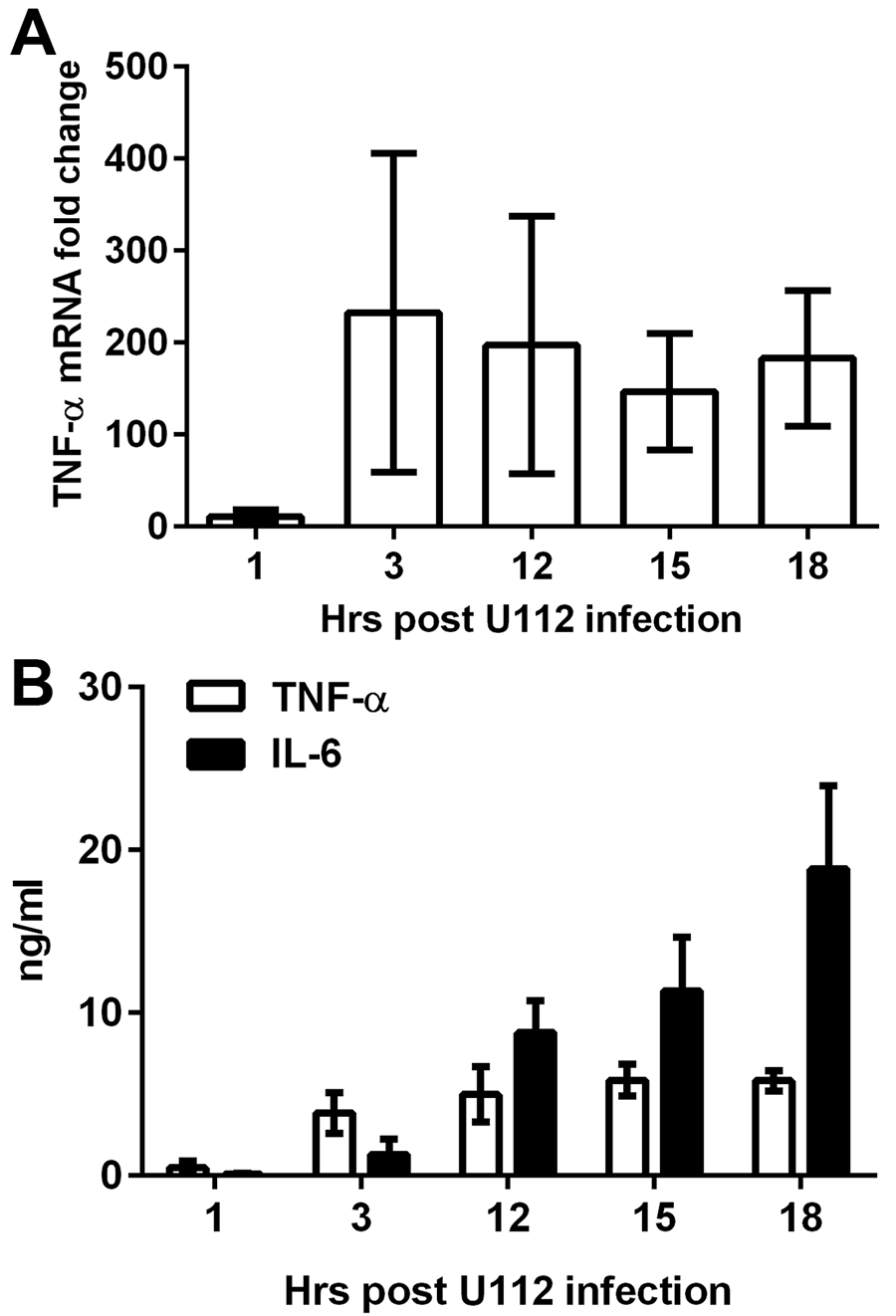

Supplement: Figure S3 — F. novicida induces secretion of proinflammatory cytokines. A–B. MDMs were infected with F. novicida strain U112 at an MOI of 100∶1. At the indicated time points, TNFα mRNA upregulation was quantified by qRT-PCR (A) and secretion of TNFα and IL-6 into the extracellular medium was quantified by ELISA (B). Data shown in each graph are the mean ±SEM of three independent experiments. (TIF) [file pone.0109525.s003.tif]
